# Gut microbiota contributes to gestational diabetes mellitus by interfering with bile acid metabolism and resistin

**DOI:** 10.3389/fcimb.2026.1675560

**Published:** 2026-02-16

**Authors:** Junhua Huang, Yujie Zhang, Wei Zheng, Guanghui Li

**Affiliations:** Department of Nutrition and Endocrinology and Metabolism, Beijing Obstetrics and Gynecology Hospital, Capital Medical University, Beijing Maternal and Child Health Care Hospital, Beijing, China

**Keywords:** Akkermansia, Bilophila, chenodeoxycholic acid, cholic acid, deoxycholic acid, Faecalibaculum, gestational diabetes mellitus, resistin

## Abstract

**Introduction:**

Gestational diabetes mellitus (GDM) affects 6% to 15% of pregnancies globally, as a severe metabolic disorder that impairs offspring health. Mounting evidence highlights the critical role of gut microbiota in metabolic regulation, yet the causal relationship between gut microbiota and GDM pathogenesis remains unclear. This study aimed to clarify this causal link and explore the underlying mechanisms.

**Methods:**

An innovative human microbiota transplantation approach was adopted. Gut microbiota from GDM patients was transplanted into antibiotic-treated C57BL/6J mice. 16S rRNA sequencing was used to analyze the structural changes of gut microbiota in recipient mice, and metabolomics was employed to detect changes in circulating bile acid levels. For mechanism exploration, Luminex assay was used to detect multiple inflammatory factors, enzyme-linked immunosorbent assay (ELISA) was applied to measure lipopolysaccharide (LPS) levels, and Western blot (WB) was utilized to determine the expression of intestinal barrier protein.

**Results:**

Transplantation of gut microbiota from GDM patients directly induced glucose intolerance in pregnant antibiotic-treated C57BL/6J mice. 16S rRNA sequencing showed significant structural reorganization of the gut microbiota in GDM microbiota recipients, characterized by decreased abundance of Lachnospiraceae_*FCS020*_group and increased abundance of Akkermansia, Faecalibaculum, and Bilophila. These microbiota dysregulations led to reduced expression of the intestinal barrier protein Claudin-1, elevated serum lipopolysaccharide (LPS) levels, and increased resistin and matrix metalloproteinase 9 (MMP-9) levels. Metabolomic analysis revealed decreased circulating primary bile acids (cholic acid [CA] and chenodeoxycholic acid [CDCA]) and secondary bile acid deoxycholic acid (DCA). Correlation analysis indicated a positive correlation between Faecalibaculum and DCA, CDCA, as well as resistin. DCA and CDCA were significantly negatively correlated with HOMA-IR, while resistin was significantly positively correlated with GTT-AUC, FINS, and HOMA-β%.

**Conclusion:**

These findings suggest that the imbalance in bile acid metabolism and mild inflammatory response caused by dysregulated gut microbiota is an adjustable environmental driving factor in the pathophysiological process of GDM.

## Graphical Abstract

**Figure:**
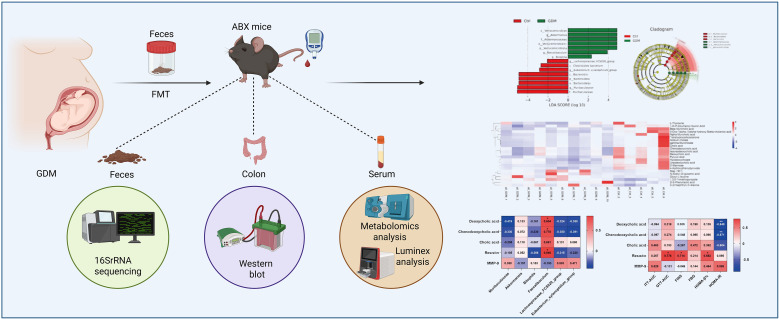
In this study, fecal microbiota transplantation (FMT) was employed to transfer the gut microbiota from patients with gestational diabetes mellitus (GDM) into antibiotic-treated mice. Techniques including 16S rRNA sequencing, liquid chromatography–tandem mass spectrometry (LC–MS/MS) analysis, Luminex-based inflammatory factor detection, ELISA, and Western blot were also applied to investigate the associations among gut microbiota, metabolomics, and inflammatory factors in GDM model mice.

## 1 Introduction

Gestational diabetes mellitus (GDM), a metabolic disorder characterized by glucose intolerance first diagnosed during pregnancy (Wang et al., 2022; Sweeting et al., 2024), poses significant risks to maternal and neonatal health (Ye et al., 2022). While its pathogenesis remains incompletely understood, emerging evidence highlights the interplay of gut microbiota dysbiosis (Medici Dualib et al., 2021), microbial metabolite perturbations (Wang et al., 2020; Ye et al., 2023; Susarla et al., 2024), and low-grade inflammation (McElwain et al., 2021) as potential contributors to GDM development. The gut microbiota, a dynamic ecosystem, regulates host metabolism and immunity through bidirectional interactions, with microbial-derived metabolites such as bile acids (Wu et al., 2023) and inflammatory mediators implicated in insulin resistance and β-cell dysfunction (Mao et al., 2024). Notably, dysregulated fecal metabolomics profiles (Gao et al., 2022; Lin et al., 2024) and elevated pro-inflammatory cytokines have been observed in GDM (McElwain et al., 2021; Zgutka et al., 2024), yet the mechanistic links between gut microbiota composition, metabolite signaling, and inflammatory pathways in this context remain underexplored.

Current research on GDM often focuses on isolated aspects of microbiota, metabolites, or inflammation, lacking a holistic approach to unravel their synergistic roles. And most clinical studies are limited by observational designs, making it difficult to distinguish whether gut microbiota dysbiosis is a cause or a secondary manifestation of GDM (Ye et al., 2023). To address this gap, our study employs a novel experimental model involving fecal microbiota transplantation (FMT) from GDM patients to antibiotic-treated C57BL/6J mice, aiming to recapitulate the gut microbial environment associated with GDM. By systematically analyzing the transplanted microbiota structure, serum metabolite profiles, and inflammatory markers in recipient mice, this work seeks to elucidate how gut dysbiosis drives metabolic and inflammatory dysregulation in GDM. The integration of multi-omics data (microbiota, metabolomics, and cytokine analysis) provides a comprehensive framework to dissect the crosstalk among microbial communities, their metabolic outputs, and host inflammatory responses.

## 2 Methods

### 2.1 Animal models and experimental design

All experimental procedures involving animals were reviewed and approved by the Institutional Animal Care and Use Committee of Beijing Huafukang Biotechnology Co., Ltd. (Protocol No. HFK-AP-20230506), in compliance with the National Institutes of Health guidelines for laboratory animal welfare. Female C57BL/6J mice (n=120; 4 weeks old; mean body weight 15.16 ± 1.23 g) were obtained from the specific pathogen-free (SPF) facility of Beijing Huafukang Biotechnology Co., Ltd. Animals were acclimatized for 7 days under controlled environmental conditions (22-25 °C, 40-60% humidity, 12:12 light-dark cycle) with ad libitum access to autoclaved feed and sterile water. Following acclimatization, mice were randomly allocated into six experimental groups (n=20/group) using a computer-generated randomization sequence (SPSS Statistics 26.0, IBM Corp.). Group identification was maintained through a unique toe-clipping numbering system. The experimental design comprised three gestational timepoints with comparative interventions:

First trimester groups: GDM-1st (FMT from GDM patients) *vs* Ctrl-1st (FMT from healthy controls)

Second trimester groups: GDM-2nd *vs* Ctrl-2nd

Third trimester groups: GDM-3rd *vs* Ctrl-3rd

To establish antibiotic-pretreated (ABX) models, all mice received daily oral gavage of a broad-spectrum antibiotic cocktail (200 µL/day) for 5 consecutive days. The antibiotic regimen consisted of:

Ampicillin (100 mg/kg; Genview, USA; Cat# AA022)

Vancomycin (50 mg/kg; Solarbio, China; Cat# V8050)

Metronidazole (100 mg/kg; Solarbio, China; Cat# IM0230)

Neomycin (100 mg/kg; Genview, USA; Cat# AN214)

Amphotericin B (1 mg/kg; Genview, USA; Cat# AA021)

FMT procedures were initiated 24 hours post-antibiotic treatment. Fresh fecal samples from donor cohorts were processed under anaerobic conditions (85% N_2_, 10% H_2_, 5% CO_2_) to prepare bacterial suspensions (50 mg feces/mL PBS). Recipient mice received daily FMT (200 µL) via oral gavage for 21 consecutive days until sexual maturation (9 weeks old).

For mating, nulliparous females were paired with proven breeder males at a 2:1 ratio. Vaginal plugs were monitored daily through visual inspection. Gestational day 0.5 (GD0.5) was designated upon plug detection, at which point dams were individually housed. Maintenance FMT was administered every 72 hours throughout gestation until parturition. Body mass measurements were recorded every 48 hours using calibrated electronic balances (± 0.01 g precision).

### 2.2 Fecal donor collection and sample preparation

Singleton pregnant women between ages 18 and 40, with complete medical records, regular prenatal examination and who delivered at Beijing Obstetrics and Gynecology Hospital, were included in the candidate population cohort after excluding the following criteria: (1) Those with gestational hypertension, diabetes, hyperlipidemia, thyroid disease, severe liver and kidney dysfunction, cardiovascular disease, intestinal diseases (constipation and diarrhea, intestinal flora disorder, etc.), and infectious diseases (hepatitis, pulmonary tuberculosis, etc.). (2) Those taking probiotics, microecological agents, and other drugs that may affect intestinal microorganisms. (3) Patients who used antibiotics within the previous 3 months. Stool samples were collected from pregnant women during their first (8–12 gestational weeks), second (24–28 gestational weeks), and third trimesters (30–34 gestational weeks) of pregnancy, and then immediately frozen in a − 80 °C refrigerator until use in subsequent experiments (**Supplementary Table 1**).

After a 75g oral glucose tolerance test (OGTT) at 24–28 weeks, the subjects were placed into two groups-a GDM group and a normal control group. GDM was diagnosed if any value reached or exceeded the cut-off value of 5.1 mmol/L for fasting, 10.0 mmol/L for 1 hour, and 8.5 mmol/L for 2 hours.

Fecal samples were collected from 42 GDM pregnant women and 42 healthy pregnant women in the control group. The samples were suspended with phosphate buffer solution (PBS, No. BF0011, Beijing Dingguo Changsheng Biotechnology Co., LTD., China) in a volume ratio of 1:5 under anaerobic conditions. After vortexing and settling for 10 minutes, we removed larger particle precipitates and collected the supernatant for FMT gavage.

### 2.3 Glucose tolerance test and insulin tolerance test of mice

On GD13.5, mice were fasted for 6 h with free water intake. After disinfecting the tail with 75% alcohol, a 0.5 mm tip was cut off, the initial blood drop removed, and fasting blood glucose was tested; body weight was measured simultaneously. Mice received an intraperitoneal glucose injection (2 g/kg body weight, Genview, USA, CG063), and blood glucose was monitored at 15, 30, 60, 90 and 120 min post-injection. Blood glucose curves were drawn for each group, with AUC calculated for inter-group glucose tolerance comparison.

At GD15.5, mice were fasted for 4 h, then their disinfected tail tip was slightly excised to measure fasting blood glucose (0 min) and body weight. Intraperitoneal insulin injection (0.5 U/kg body weight, Genview, USA, FI174) was performed, and blood glucose levels were determined at 15, 30, 60, 90 and 120 min post-injection. Corresponding blood glucose curves and AUC values were obtained for group analysis.

### 2.4 Measurement of fasting blood glucose and fasting serum insulin

On GD18.5, mice were fasted for 12 hours while having free access to drinking water. After disinfecting the tail with 75% medical ethanol, a 0.5-mm nick was made, and the first blood drop was wiped away. FBG levels were detected by allowing blood to adhere to test strips and using a Roche blood glucose meter. Orbital blood was then harvested to assess fasting insulin levels, with FINS quantified using a mouse ultrasensitive insulin ELISA kit (No. PI602, Beyotime Biotech. Inc., China). Using the obtained FBG and FINS data, insulin sensitivity and islet β-cell function were calculated with the formulas below:


HOMA−IR=(FBG×FINS)/22.5

HOMA−β%=(20×FINS)/(FBG−3.5)

### 2.5 Detection of serum inflammatory cytokine levels in mice using luminex technology

We used the Luminex Mouse Discovery Assay (10-Plex) (No. LXSAMSM-10, Bio-Techne R&D systems, No. USA) to detect inflammatory cytokine levels in serum samples from FMT model mice according to the manufacturer’s instructions. The assay detected tumor necrosis factor-alpha (TNF-α), growth differentiation factor 15 (GDF-15), C-C motif chemokine ligand 2 (CCL2), and interleukin-1beta (IL-1β), interleukin-6 (IL – 6), resistin, adiponectin, CXC chemokine ligand-10 (CXCL10), matrix metalloproteinases 9 (MMP-9), and C - reactive protein (CRP). Three multiple wells were made for each sample. The plate was read on Luminex 200 instrument and the data acquisition and analysis were conducted using the Luminex xPONENT software.

### 2.6 Mice gut microbiome analysis

Mouse fecal genomic DNA was extracted with the Tiangen Fecal Genomic DNA Extraction Kit, followed by spectrophotometric assessment of concentration and purity. Barcoded primers (341F/806R) were used to amplify the 16S rRNA V3-V4 regions. PCR products were fluorometrically quantified, gel-validated, and magnetically purified before library preparation with the NEB Next^®^ Ultra™ DNA Library Prep Kit. Sequencing was conducted on the Illumina NovaSeq 6000 platform (2×250 bp paired-end).

Bioinformatic processing involved FLASH v1.2.11 read merging, Trimmomatic v0.39 quality filtering, UCHIME chimera removal, and DADA2 denoising for ASV generation. Taxonomic assignment was performed against the SILVA 138 database (70% confidence). Post-rarefaction, α-diversity (Shannon, Simpson, Chao1) and β-diversity (weighted UniFrac) were calculated in QIIME2 v2021.4, with PCoA visualizing community differences. LEfSe (LDA > 2) identified differential taxa, and Tax4Fun predicted functional potential via KEGG annotation.

### 2.7 Metabolite extraction and LC-MS/MS analysis

Serum metabolites were extracted using pre-chilled 80% methanol, followed by ice incubation, centrifugation, and dilution to a final concentration of 53% methanol before LC-MS/MS analysis. The analysis was performed using an ExionLC™ AD system coupled with a QTRAP^®^ 6500+ mass spectrometer. Separation was achieved on an Xselect HSS T3 column with gradient elution in both positive and negative ion modes. Metabolites were identified and quantified using the Novogene in-house database based on Q1/Q3 ions, retention time, declustering potential (DP), and collision energy (CE). Data processing, including peak integration and correction, was conducted using SCIEX OS software.

Metabolites were annotated using the KEGG, HMDB, and Lipidmaps databases. Differential metabolites were screened based on variable importance in projection (VIP) > 1, P-value < 0.05 (t-test), and fold change (FC) ≥ 2 or ≤ 0.5. Visualization was performed using volcano plots and clustering heatmaps. Metabolic pathway enrichment analysis was conducted using the KEGG database, with significance determined by the ratio of differential metabolites in the pathway (x/n) compared to the background (y/N) and a P-value < 0.05.

### 2.8 Western blot analysis

Proteins were extracted from tissue samples using RIPA lysis buffer supplemented with phenylmethanesulfonyl fluoride (PMSF; Beyotime Institute of Biotechnology, China). Protein concentration was quantified using an Enhanced BCA Protein Assay Kit (Beyotime Institute of Biotechnology, China). Equal amounts of protein were separated by SDS-PAGE on 10% or 15% gels (Solarbio Science & Technology Co., Ltd., China) and then transferred onto polyvinylidene fluoride (PVDF) membranes (Merck Millipore, USA). The membranes were blocked with 5% non-fat dry milk for 2 hours at room temperature, followed by incubation overnight at 4 °C with the following primary antibodies: anti-Occludin (1:500, ab235986, Abcam, UK), anti-Claudin-1 (1:25, ab15098, Abcam, UK), anti-ZO-1 (1:1000, #13663, Cell Signaling Technology, USA), and anti-GAPDH (1:1000, AF1186, Beyotime Institute of Biotechnology, China). After washing three times with TBST (Solarbio Science & Technology Co., Ltd., China), the membranes were incubated with corresponding horseradish peroxidase (HRP)-conjugated secondary antibodies (goat anti-rabbit or goat anti-mouse IgG; 1:1000; Beyotime Institute of Biotechnology, China) for 1 hour at room temperature. Protein bands were visualized using a chemiluminescent substrate (Merck Millipore, USA) and imaged with a ChemiDoc™ XRS+ system (Bio-Rad Laboratories, USA).

### 2.9 Measurement of serum LPS levels

Lipopolysaccharide (LPS) concentrations in mouse serum were quantified using a specific commercial ELISA kit (Mouse Lipopolysaccharides, LPS ELISA Kit, SP14143, Wuhan Spbio Biotechnology Co., Ltd., China). All assay procedures were performed strictly following the manufacturer’s protocol.

### 2.10 Statistical analysis

The study results were presented either as the mean ± standard error of the mean (SEM) or as the median (first quartile, third quartile). For intergroup comparisons, distinct statistical tests were employed based on the characteristics of the variables. Specifically, for variables that were normally distributed and had equal variances, an independent samples t-test was utilized. In contrast, when dealing with variables that were non-normally distributed or had unequal variances, the Mann-Whitney U test was applied. Regarding longitudinal comparisons across different gestational time points, a two-way analysis of variance (ANOVA) with Bonferroni correction was carried out to account for multiple comparisons. To evaluate the monotonic relationships between variables, Spearman’s rank correlation coefficient was calculated. All statistical analyses were performed using IBM SPSS Statistics 26.0 (IBM Corp.) and GraphPad Prism 9.0 (GraphPad Software). Statistical significance was set at a two - tailed α - level of 0.05.

## 3 Results

### 3.1 Feces from second trimester of GDM patients induce glucose metabolic disturbances in a murine model

**Figure 1:**
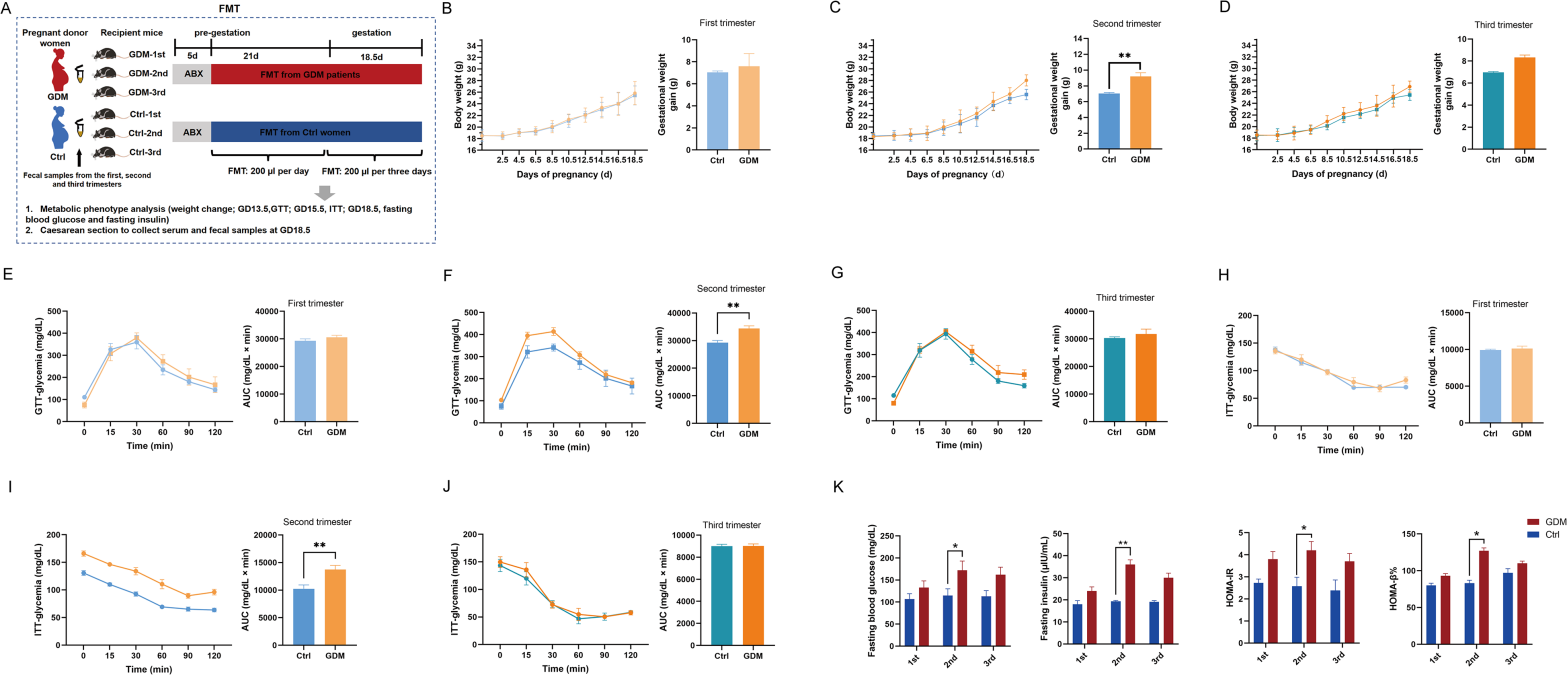
FMT presented characteristics of the GDM phenotype. **(A)** Schematic diagram of animal experiment scheme. **(B–D)** Weight changes during pregnancy. **(E–G)** GTT and area under the curve **(AUC)** at GD13.5. **(H–J)** ITT and area AUC at GD15.5. **(K)** FBG, FINS, HOMA-β% and HOMA-IR at GD18.5. The data were expressed as means ± SME. *p<0.05, **p<0.01,*** p<0.001. FMT, fecal microbiota transplantation; GDM, gestational diabetes mellitus.

To systematically evaluate GDM-associated phenotypes, FMT was administered to six experimental groups: GDM-1st *vs* Ctrl-1st; GDM-2nd *vs* Ctrl-2nd; and GDM-3rd *vs* Ctrl-3rd (**Figure 1A**). Longitudinal monitoring revealed that mice receiving second-trimester GDM-derived FMT (GDM-2nd group) displayed significant metabolic perturbations compared to gestational age-matched controls. At gestational day (GD) 18.5, the GDM-2nd group exhibited elevated absolute body weight (28.02 ± 3.41 g *vs* 25.58 ± 2.76 g; p=0.030) and exaggerated gestational weight gain (9.67 ± 0.48 g *vs* 7.20 ± 0.12 g; p=0.002), whereas no intergroup differences were observed in the first- or third-trimester FMT groups (**Figures 1B–D**).

Metabolic profiling through intraperitoneal glucose tolerance tests (GTT) and insulin tolerance tests (ITT) demonstrated impaired glycemic regulation specifically in the GDM-2nd group. Quantitative analysis of area under the curve (AUC) revealed significantly elevated glucose excursions during GTT (34399.00 ± 518.54 vs 29282.00 ± 631.47; p=0.0019) and blunted insulin responses during ITT (13726.00 ± 593.90 vs 10235.00 ± 602.33; p=0.0042) compared to Ctrl-2nd mice (**Figures 1E–J**). Subsequent biochemical analyses confirmed metabolic dysregulation in the GDM-2nd group, with elevated fasting blood glucose (FBG: 172.00 ± 4.70 mg/dL *vs* 115.00 ± 3.35 mg/dL; p=0.030), increased fasting insulin (FINS: 36.00 ± 0.48 μIU/mL *vs* 19.30 ± 0.09 μIU/mL; p=0.003), and concomitant rises in homeostasis model assessment indices for insulin resistance (HOMA-IR: 4.20 ± 0.09 *vs* 2.58 ± 0.09; p=0.011) and β-cell function (HOMA-β%: 127 ± 0.92 *vs* 83.00 ± 0.89; p=0.034) (**Figure 1K**).

These findings establish a critical temporal association between second-trimester microbial dysbiosis and GDM pathogenesis. The selective emergence of hyperglycemia, insulin resistance, and excessive weight gain exclusively in the GDM-2nd group implicates mid-gestation as a vulnerable window for microbiota-mediated metabolic dysregulation. The phenotypic recapitulation of human GDM hallmarks in this murine model underscores the potential causal role of gut microbiota restructuring in disease progression.

### 3.2 GDM-associated gut microbiota remodeling drives metabolic dysregulation in murine models

**Figure 2:**
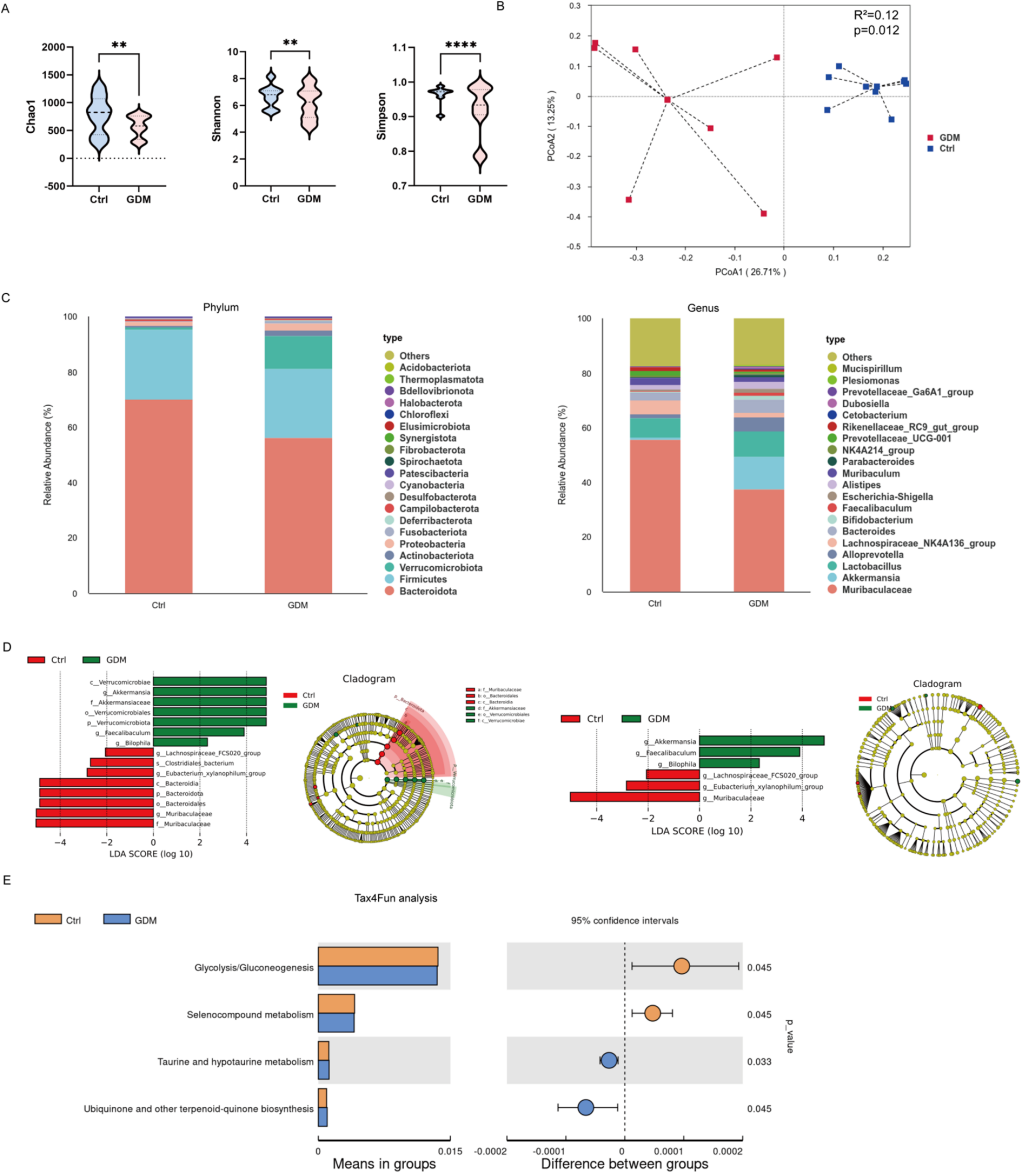
Dysbiosis of gut microbiota in mice with a GDM model. **(A)** α-diversity indices (Chao1, Shannon, and Simpson). **(B)** β-diversity, calculated based on the Bray - Curtis dissimilarity metric. **(C)** Histogram of species abundance between two groups at phylum and genus taxonomic level. **(D)** LEfSe analysis, presented as an LDA Score bar chart and a Cladogram all taxonomic levels and at the genus level, LDA>2. **(E)** Relative abundance of Kyoto Encyclopedia of Genes and Genomes (KEGG) pathways in the gut microbiota, predicted by Tax4fun. **p<0.01,**** p< 0.0001. GDM, gestational diabetes mellitus.

Following the successful establishment of GDM phenotypes in mice through FMT from second-trimester GDM patients, we performed comprehensive characterization of intestinal microbial communities in these GDM-FMT models using 16SrRNA sequencing. Significant alterations in α-diversity metrics were observed between groups, with the GDM group demonstrating reduced microbial richness (Chao1 index: p=0.001) and diminished diversity (Shannon index: p=0.003; Simpson index: p<0.0001) compared to controls (**Figure 2A**). β-diversity analysis via bray-curtis dissimilarity metrics revealed distinct clustering patterns between groups (PERMANOVA, R²=0.12, p=0.012), indicating fundamental differences in microbial community structure (**Figure 2B**).

Taxonomic profiling demonstrated marked compositional shifts at both phylum and genus levels. At the phylum level, the GDM group exhibited relative enrichment of Verrucomicrobiota (11.73% *vs* 0.85% in controls) concurrent with depletion of Bacteroidota (56.46% *vs* 70.15%).At the genus level, the proportions of Akkermansia in the GDM and control groups were 11.71% and 0.80% respectively, while the proportions of Muribaculaceae in the two groups were 37.78% and 55.80% respectively (**Figure 2C**). Linear discriminant analysis effect size (LEfSe) identified specific operational taxonomic units (OTUs) differentiating the groups, with three genera showing significant elevation in GDM models: Akkermansia (LDA = 4.85, p=0.02), Faecalibaculum (LDA = 3.90, p=0.030), and Bilophila (LDA = 2.32, p=0.040). Conversely, control mice displayed enrichment of butyrate-producing taxa including Lachnospiraceae_FCS020_group (LDA = 2.06, p=0.040), Eubacterium_xylanophilum_group (LDA = 2.84 p=0.040), and Muribaculaceae (LDA = 5.03, p=0.020) (**Figure 2D**).

Functional prediction using Tax4Fun (v1.0) revealed distinct metabolic pathway profiles. Control-associated microbiota showed increased potential for glycolysis/gluconeogenesis (KEGG pathway ko00010) and selenocompound metabolism (ko00450). The GDM microbiome exhibited enhanced predictive capacity for taurine/hypotaurine metabolism (ko00430) and ubiquinone biosynthesis (ko00130) (**Figure 2E**). These functional disparities suggest that GDM-associated microbial communities may disrupt host metabolic homeostasis through modulation of bile acid metabolism (Collins et al., 2023; Sun et al., 2024) and mitochondrial electron transport processes (Wang et al., 2024).

### 3.3 Metabolomics reveals altered bile acid synthesis and transport in GDM mice with gut microbiota dysbiosis

**Figure 3:**
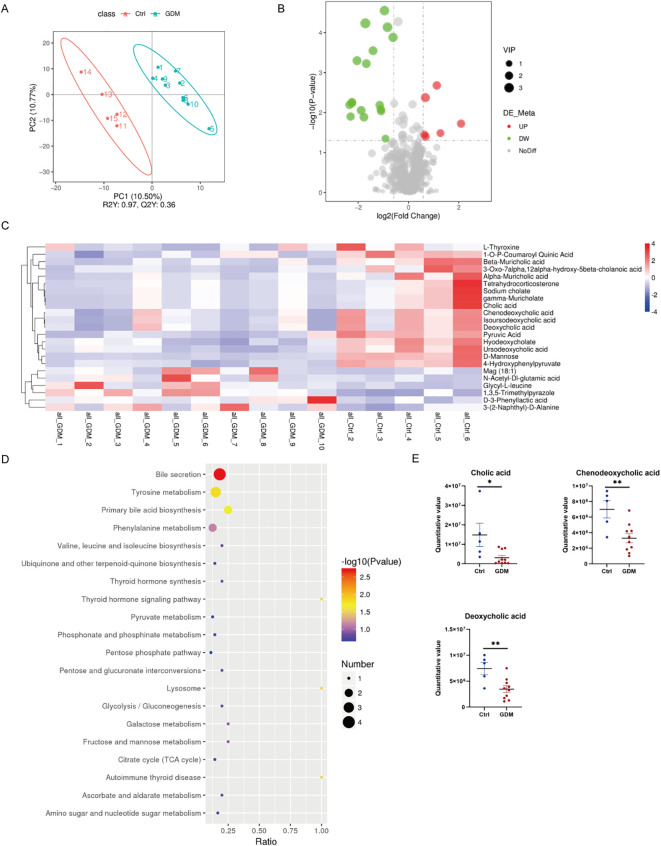
Serum metabolomics analysis. **(A)** Partial least squares discriminant analysis (PLS - DA). **(B)** Volcano plot of differential metabolites when comparing the GDM group with the control group. **(C)** Clustering heatmap of differential metabolites between the two groups. **(D)** Bubble plot for KEGG enrichment analysis. **(E)** Bar chart of intergroup comparison of CA, CDCA, and DCA metabolites. *p < 0.05, ** p <0.01. GDM, gestational diabetes mellitus; CA, cholic acid; CDCA, chenodeoxycholic acid; DCA, deoxycholic acid.

Based on the finding that gut microbiota dysbiosis may affect bile acid metabolism, we performed metabolomics analysis on the serum of model mice using ultra-high-performance liquid chromatography-tandem mass spectrometry (UHPLC-MS/MS). Partial least squares discriminant analysis (PLS-DA) showed that the samples from the GDM group and the control group were significantly separated in the principal component space (R²Y = 0.97, Q²Y= 0.36), indicating systematic differences in metabolic characteristics between the two groups (**Figure 3A**). A total of 23 significantly changed metabolites were identified by screening differential metabolites (thresholds: VIP > 1.0, FC > 1.2 or < 0.833, p < 0.05). Among them, 6 were up-regulated in the GDM group (such as Glycyl - L - leucine, Monoacylglycerol MAG 18:1) and 17 were down-regulated (**Figures 3B, C**). The hierarchical clustering heatmap further showed that both primary bile acids (cholic acid [CA], chenodeoxycholic acid [CDCA]) and secondary bile acids (deoxycholic acid [DCA]) were significantly decreased in the GDM group (Δlog2FC = - 1.09~ - 2.25, p < 0.01). Meanwhile, 3 - Phenyllactic acid, a metabolic intermediate of the precursor of taurine - conjugated bile acids (such as taurocholic acid), was significantly increased (Δlog2FC = 0.63, p = 0.035) (**Figure 3C**).

KEGG pathway enrichment analysis (FDR < 0.05) showed that the differential metabolites were significantly enriched in the bile acid secretion pathway (ko04976, p = 0.002). The key - node metabolites CA (p=0.019), CDCA (p=0.006), and DCA (p=0.006) in this pathway decreased significantly in the GDM group as opposed to the control group (p < 0.05) (**Figures 3D, E**). Notably, Tetrahydrocorticosterone, a metabolite related to the key transporter regulating the enterohepatic circulation of bile acids [such as the ileal apical sodium - dependent bile acid transporter [ASBT] (Caballero-Camino et al., 2023)], was also significantly decreased (Δlog2FC = -2.305, p = 0.013) (**Figure 3C**).

### 3.4 Gut microbiota dysbiosis may influence bile acid and glucose metabolism by impairing intestinal barrier function and inducing low-grade inflammation

**Figure 4:**
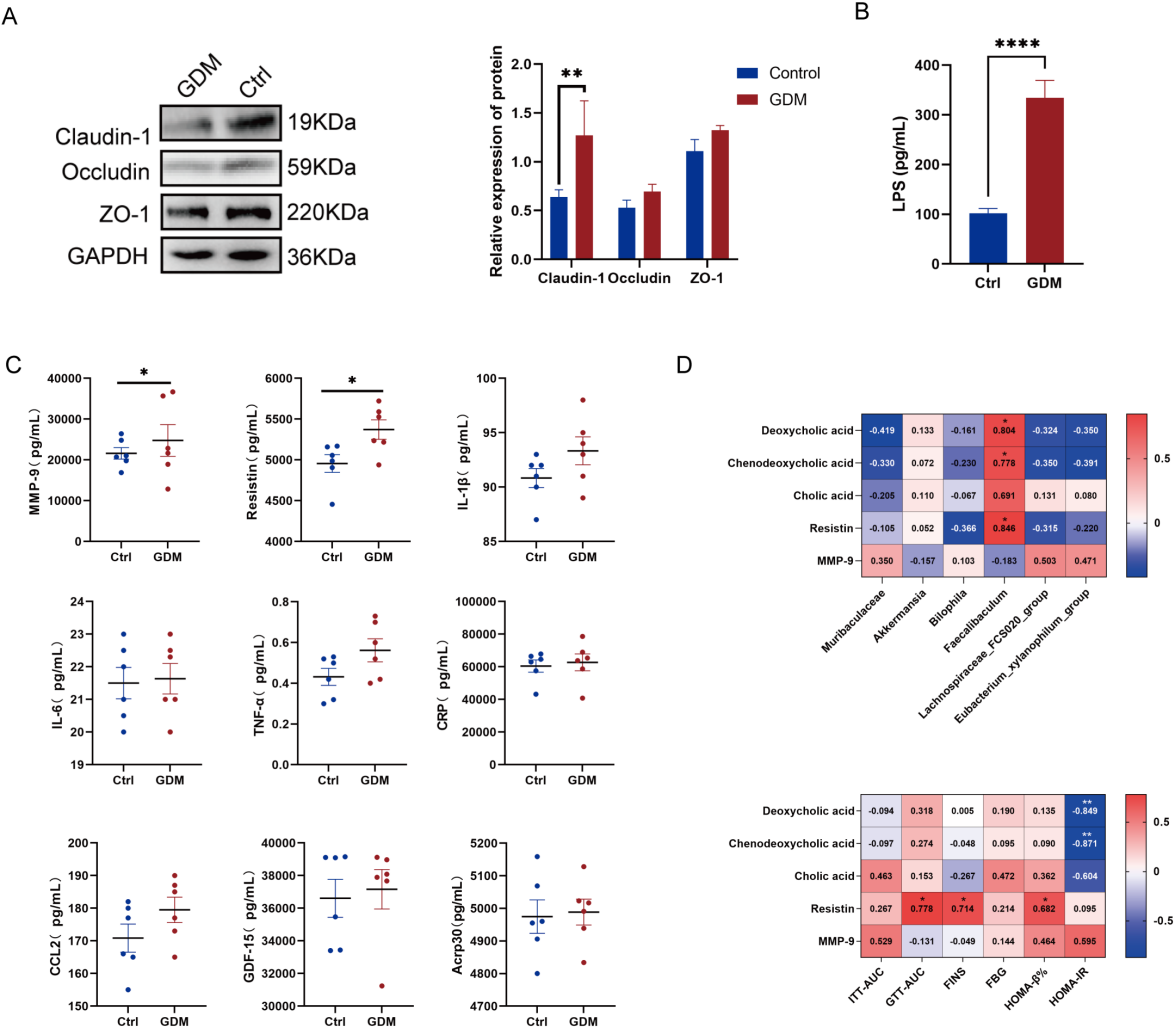
Disruption of gut microbiota damages the intestinal barrier and induces low-grade inflammation. **(A)** Western blot for Claudin-1, Occludin and ZO-1. **(B)** ELISA for LPS. **(C)** Serum contents of 9 inflammatory factors. **(D)** Correlation analysis of differential metabolites related to bile acid metabolism, resistin, MMP9, and insulin resistance assessment indicators. *p < 0.05, **p < 0.01, ****p<0.0001.

Based on the aforementioned results of gut microbiota dysbiosis and abnormal bile acid metabolism, combined with literature evidence (Ma et al., 2020; Ma et al., 2024), we speculated that gut microbiota dysbiosis might affect bile acid and glucose metabolism by impairing intestinal barrier function. To test this hypothesis, we next examined the expression of intestinal barrier proteins in colon tissues and the serum lipopolysaccharide (LPS) levels in both groups of mice. We found that the expression of Claudin-1 protein in colon tissues of the GDM group was significantly reduced, while the other two barrier proteins, Occludin and ZO-1, also showed a decreasing trend (**Figure 4A**). Additionally, serum LPS levels in the GDM group were significantly elevated (334.20 ± 34.20 pg/mL *vs* 113.00 ± 9.60 pg/mL; p<0.0001) (**Figure 4B**). These results suggest impaired intestinal barrier function in GDM group mice.

To investigate whether LPS entry into the bloodstream due to barrier damage triggers an inflammatory response, we used Luminex technology to measure the levels of 10 inflammatory cytokines in the serum of both groups. These cytokines included tumor necrosis factor-α (TNF-α), growth differentiation factor 15 (GDF-15), C-C motif chemokine ligand 2 (CCL2), interleukin-1β (IL-1β), interleukin-6 (IL-6), resistin, adiponectin, CXC chemokine ligand 10 (CXCL10), matrix metalloproteinase 9 (MMP-9), and C-reactive protein (CRP). Unfortunately, we were unable to detect the actual concentration of CXCL10, as its levels in each sample were too low, with fluorescence values below those of the background wells. By comparing the serum inflammatory cytokine concentrations between the two groups, we found that MMP-9 (22248.50 [17385.75, 35929.25] pg/mL *vs*. 20873.50 [19062.25, 25229.00] pg/mL, p = 0.046) and resistin (5381.50 [5147.50, 5624.00] pg/mL *vs*. 5021.50 [4793.30, 5161.00] pg/mL, p = 0.046) levels were significantly higher in the GDM group than in the control group (**Figure 4C**), suggesting that intestinal barrier dysfunction triggered a mild systemic inflammatory response.

We further conducted a correlation analysis and found that Faecalibaculum was positively correlated with DCA and CDCA, and with resistin. DCA and CDCA were significantly negatively correlated with HOMA-IR, while resistin was significantly positively correlated with GTT-AUC, FINS, and HOMA-β%. (**Figure 4D**).

## 4 Discussion

The findings of this study provide compelling evidence that gut microbiota dysbiosis in GDM plays a critical role in metabolic dysregulation, including impaired glucose homeostasis and altered bile acid metabolism. By transplanting fecal microbiota from GDM patients into antibiotic-treated C57BL/6J mice, we observed significant metabolic disturbances, such as hyperglycemia, increased gestational weight gain, and reduced levels of primary and secondary bile acids (CA, CDCA, DCA). Intestinal barrier damage causes LPS to enter the bloodstream, leading to an increase in the levels of some inflammatory cytokines in the serum. These findings provide critical insights into the potential role of gut microbiota in mediating metabolic dysregulation during GDM and highlight a complex interplay between microbial communities, host metabolism, and inflammation.

Our study investigated the differential effects of FMT from GDM donors at early, mid, and late pregnancy stages on metabolic phenotypes in mouse models. We observed that only mice receiving FMT from mid-pregnancy GDM donors developed significant metabolic abnormalities, while those receiving early or late-pregnancy FMT did not exhibit notable differences. This stage-specific effect aligns with clinical patterns and may reflect dynamic host-microbe interactions during pregnancy. Clinically, GDM is typically diagnosed between 24–28 weeks of gestation (mid-pregnancy), as glucose intolerance becomes most pronounced during this period (Sweeting et al., 2022). In early pregnancy, most women who later develop GDM still exhibit normal glucose metabolism (Simmons et al., 2023), suggesting that gut microbiota dysbiosis may not yet be fully established or functionally impactful (Ma et al., 2020; Ma et al., 2024; Wu et al., 2025). Additionally, hormonal shifts—especially the early pregnancy rise in human chorionic gonadotropin and the ovarian secretion of progesterone and estrogen before placental maturation (Armistead et al., 2020)—create a fluctuating metabolic environment that may limit the stabilization of a “GDM microbiota phenotype.” Consequently, microbial colonization during this phase may be too transient or unstable to consistently transfer diabetogenic traits via FMT. Mid-pregnancy represents a critical window for GDM pathogenesis. Hormonal changes peak during this period, interacting with the gut microbiota to promote insulin resistance and low-grade inflammation (Hasain et al., 2020). The microbiota composition also stabilizes somewhat, allowing GDM-associated pathobionts to exert more pronounced metabolic effects when transplanted. Moreover, metabolite accumulation—such as reduced short-chain fatty acids (Chen et al., 2022; Wang et al., 2022) and elevated LPS (Zhang et al., 2021)—may reach a threshold capable of inducing systemic insulin resistance in recipients. In late pregnancy, although GDM-related dysbiosis may persist, clinical interventions (e.g., dietary modifications or physical activity) often ameliorate glucose levels and partially restore microbial balance after diagnosis (Benham et al., 2023). This may explain why FMT from late-gestation GDM donors—who may have received glycemic management—did not induce significant metabolic disturbances in mice. Furthermore, physiological adaptations in late pregnancy (e.g., immune modulation and metabolic prioritization for parturition) (Hamburg-Shields and Mesiano, 2024) could attenuate the pro-diabetic influence of microbiota.

The altered α- and β-diversity in GDM-transplanted mice mirrors observations in human GDM cohorts, where reduced microbial diversity and imbalanced taxa are linked to insulin resistance and glucose intolerance (Yan et al., 2022). Notably, the enrichment of Akkermansia, Faecalibaculum, and Bilophilain GDM mice aligns with previous reports linking these genera to metabolic dysfunction (Medici Dualib et al., 2021; Rold et al., 2022). Akkermansia, in particular, exhibits a context-dependent role—associated both with metabolic improvements and with inflammatory conditions depending on host physiology and diet (Qin et al., 2022). While Akkermansia muciniphilais often linked to better metabolic health in obesity and type 2 diabetes (T2DM) (Wu et al., 2022), its overrepresentation in GDM-FMT mice may reflect a pregnancy-specific effect, likely shaped by the distinct hormonal milieu of gestation (Qi et al., 2021). The pronounced elevations in progesterone and estrogen during pregnancy can substantially reshape immune function and host-microbe interactions, potentially altering the net metabolic influence of Akkermansiain susceptible individuals (Lu et al., 2024). Furthermore, Akkermansiadid not occur in isolation but co-enriched with genera such as Faecalibaculum and Bilophila, both implicated in promoting inflammation and barrier dysfunction (Sayavedra et al., 2025). This co-occurrence within a dysbiotic consortium suggests that the overall functional output of the microbial community—rather than a single taxon—may drive metabolic disturbances in GDM.

Faecalibaculum has been implicated in bile acid metabolism and inflammation (Shen et al., 2020), and bile acid signaling is a key regulator in glucose homeostasis (Xu et al., 2023). Conversely, the depletion of Lachnospiraceae_FCS020_group and Muribaculaceae in GDM group—taxa linked to short-chain fatty acid (SCFA) production—may impair intestinal barrier integrity and exacerbate systemic inflammation (Iliev et al., 2025), further aggravating IR. The significant reduction in serum CA, CDCA, and DCA in GDM-FMT mice underscores the critical role of bile acids in metabolic regulation. Bile acids act as signaling molecules via the farnesoid X receptor (FXR) and TGR5 receptors, modulating glucose metabolism and insulin sensitivity (Kriaa et al., 2022). The observed negative correlations between DCA/CDCA and HOMA-IR suggest that diminished bile acid levels may impair FXR/TGR5 activation, leading to hepatic gluconeogenesis and peripheral IR (Gao et al., 2022; Münzker et al., 2022). This aligns with studies showing that FMT from healthy donors restores bile acid profiles and improves insulin sensitivity in diabetic models (Zhang et al., 2020).

Resistin, an adipokine linked to IR, promotes hepatic glucose output and adipose tissue dysfunction (Li et al., 2021), while MMP-9 contributes to extracellular matrix remodeling and chronic inflammation (Wolosowicz et al., 2025). The elevated resistin and MMP-9 levels in GDM-FMT mice highlight the interplay between gut microbiota and systemic inflammation. This inflammatory response is likely due to the compromised integrity of the intestinal barrier, which is confirmed by the significant decrease in Claudin-1 expression in the colon of GDM mice, allowing LPS to enter the bloodstream. The increase in LPS in the circulation triggers innate immune activation and promotes the release of pro-inflammatory mediators. This mechanism has been supported by previous studies, which indicate that intestinal barrier dysfunction is associated with metabolic inflammation (Zhang et al., 2022).The positive correlation between resistin and GTT-AUC/HOMA-β% suggests that inflammatory mediators may directly impair pancreatic β-cell function, exacerbating hyperglycemia (Mittal et al., 2025).

This study demonstrates that FMT from patients with GDM can replicate key metabolic and inflammatory features of GDM in a mouse model. Our findings suggest that gut microbiota dysbiosis may contribute to the pathogenesis of GDM by disrupting bile acid metabolism and promoting systemic inflammation, thereby providing valuable preliminary insights into the “gut microbiota–bile acid–inflammation” axis in GDM. However, as an exploratory mechanistic study, this research has certain limitations. Although correlation analyses revealed associations between certain bacterial genera such as Faecalibaculum and bile acid levels or resistin, these associations do not establish causality. Furthermore, our study did not perform targeted interventions, such as using bile acid receptor agonists/antagonists or cytokine neutralization, to functionally validate the inferred mechanisms or dissect the precise host–microbe interactions involved. Future research should aim to move beyond correlation and establish causal relationships. Specifically, the direct role of key bacterial taxa, such as Faecalibaculum, could be elucidated through *in vitro* experiments using pure bacterial cultures on intestinal epithelial cells or through *in vivo* FMT studies combined with selective bacterial depletion or supplementation.

## 5 Conclusions

Based on the research findings, intestinal microbiota dysbiosis appears to be a significant driver in the pathophysiology of gestational diabetes mellitus (GDM), primarily mediated through disruptions in bile acid metabolism and the activation of inflammatory pathways. These insights suggest the potential for microbiota-targeted interventions to ameliorate metabolic disturbances associated with GDM. Further detailed mechanistic studies exploring microbial-based therapeutic strategies are warranted to translate these findings into clinical applications.

## Data Availability

The raw data supporting the conclusions of this article will be made available by the authors, without undue reservation.
