# Supplementary material for: Gut microbiota contributes to gestational diabetes mellitus by interfering with bile acid metabolism and resistin
Source: Front Cell Infect Microbiol. 2026 Feb 16;16:1675560. doi: 10.3389/fcimb.2026.1675560 (PMC12950734; doi:10.3389/fcimb.2026.1675560)
Supplement: Supplementary file 1 [file Table1.docx]

**Supplementary Materials**

**Supplementary Table 1. The Basic Characteristics of Fecal Donors**

| Maternal characteristics | Control (n=42) | GDM (n=42) | p |
| --- | --- | --- | --- |
| Age, years | 34.1±3.9 | 33.9±3.9 | 0.53 |
| Gravidity, 1st | 6 (14.29%) | 7 (16.67%) | 0.72 |
| Parity, 1st | 11 (26.20%) | 12 (28.57%) | 0.61 |
| Height, cm | 161.13±4.47 | 161.51±5.14 | 0.90 |
| Prepregnancy weight, kg | 55.75±6.90 | 60.40±7.14 | 0.02* |
| Prepregnancy BMI, kg/cm^2^ | 21.50±2.72 | 23.03±3.95 | 0.04* |
| Gestational age, weeks | 39 (38-39) | 39 (38-39) | 0.34 |

GDM, gestational diabetes mellitus; BMI, body mass index.*p<0.05.
